# Precarious Manhood Beliefs Are Positively Associated with Erectile Dysfunction in Cisgender Men

**DOI:** 10.1007/s10508-023-02640-4

**Published:** 2023-06-23

**Authors:** Andreas Walther, Timothy Rice, Lukas Eggenberger

**Affiliations:** 1 Department of Clinical Psychology and Psychotherapy, Psychological Institute, University of Zurich, Binzmühlestrasse 14, 8050 Zurich, Switzerland; 2 Department of Psychiatry, Icahn School of Medicine at Mount Sinai, New York, NY USA

**Keywords:** Precarious Manhood Beliefs, Erectile dysfunction, Masculinity, Sexual dysfunction, Sexual function

## Abstract

The notions that manhood is hard to attain, easy to lose, and needs to be proven via public action constitute precarious manhood beliefs (PMB). PMB is a new concept and it remains unclear whether and how PMB relate to erectile dysfunction (ED) in cisgender men. The ability to achieve an erection remains considered as a cornerstone of masculinity and sexual performance can be conceived as a proof of one’s masculinity. In this context, ED can be received as sexual failure and a threat to a man’s masculinity and sense of adequacy. For these reasons, the hypothesis that PMB are associated with ED warranted empirical testing. In an anonymous online survey focusing on men’s mental health conducted in German-speaking countries of Europe, 507 cisgender men (*M*_age_ = 44.2, *SD*_age_ = 15.2) completed measures on PMB, sexual function, self-stigma, social desirability, and conformity to traditional masculinity ideology (TMI). Multilinear regression analysis with stepwise introduction of relevant covariates evaluated potential associations between PMB and ED. Of a 507 cisgendered male sample, 63.1% reported an increased risk for ED based on previously established cutoff points. Elevated levels of PMB endorsement among the men predicted reduced sexual and erectile function in all models, even when accounting for relevant control variables such as age, education, self-stigma, social desirability, or conformity to TMI. Group comparisons revealed that the men suffering from ED showed higher levels of PMB endorsement but not self-stigma nor TMI relative to men without ED. PMB are significantly associated with ED. While determining causality will require further study, our results may support the hypothesis that higher levels of PMB endorsement may lead to increased tension to perform sexually, resulting in increased psychological pressure and a higher risk to develop ED.

**Supplementary Information:**

The online version contains supplementary material available at 10.1007/s10508-023-02640-4.

## Introduction

Traditional masculinity ideologies (TMI) emphasize the pursuit of status, dominance, toughness, self-reliance, and anti-femininity as characteristics of what it means to be a man (Levant & Richmond, 2016). Gender role socialization describes the fluid process through which children and adults internalize or discard the values, attitudes, and behaviors associated with femininity, masculinity, or both (O’Neil, 2013). Adherence to TMI can influence male gender role socialization through its rigid characterizations of masculinity (Gerdes et al., 2018; Levant & Richmond, 2016).

From an early age, men socially learn to evaluate their own adequacy based on their ability to behave in accordance with their gender role. Behavior outside of gendered norms commonly leads to sanctioning, which has been shown to be harsher for boys and men than girls and women (Levy et al., 1995; McCreary, 1994; Sirin et al., 2004; Vandello & Bosson, 2013; Vandello et al., 2008). Across cultures, manhood is experienced as a tenuous state which must be acquired through public action (Vandello & Bosson, 2013; Vandello et al., 2008). Men believe that it can be lost through non-conforming gendered behavior, such as showing fear or lacking courage (Vandello & Bosson, 2013; Vandello et al., 2008).

Precarious manhood theory (PMT; Vandello & Bosson, 2013; Vandello et al., 2008), proposes that masculinity is easily threatened compared to femininity. PMT identifies threats to masculinity as challenges to man's overall sense of adequacy and self-worth. Dahl and Cook (2020) advance that the self-worth of a man is composed of different self-concepts (Dahl & Cook, 2020). Most men’s self-worth appears contingent on a personal sense of masculinity as a key self-concept (Burkley et al., 2016; Wong et al., 2017).

In exploring male gender role socialization in the context of TMI and PMT, it is important to consider the impact of erectile dysfunction (ED). ED is defined as the persistent or recurrent inability to achieve or maintain an erection sufficiently strong for satisfactory intercourse (McVary, 2007). ED is the most prevalent sexual dysfunction in men, affecting over a quarter of the general male population worldwide (Kessler et al., 2019). ED reduces the quality of life of men and their partners (Kessler et al., 2019). In addition to the physiologic loss of function in ED, men with ED can experience an psychological impact. Men with ED commonly feel a loss of their own masculinity (Loe, 2001; Potts, 2000; Sand et al., 2008; Ussher et al., 2017; Zaider et al., 2012). ED can destabilize a man's sense of adequacy and positive self-worth (Dahl & Cook, 2020).

Many men experience an ED “double jeopardy”: men are first affected by the failure of erectile functioning and then by the additional psychological distress stemming from their inability to live up to sexuality-related TMI, such as beliefs that they must be sexually fit, tough, and self-reliant. Although many risk factors for ED, such as an unhealthy lifestyle, genetic or hormonal markers, medical conditions, relationship satisfaction, and intimacy motivation, exist (Allen & Walter, 2019; Walther et al., 2017), few studies have examined the relationship between men’s adherence to concepts of masculinity and their erectile functioning.

Thompson and Barnes (2013) investigated 132 men over the ages of 50 years and found that while mostly sexually healthy men renounced the notion that erectile ability is essential to a man’s masculinity, men with ED were less likely to do so. This finding occurred despite equivalent rates of overall TMI endorsement between men with and without ED. In another study concerning a sample of 116 male spinal cord injury survivors, only one out of five TMI subdimensions, termed “primacy of work,” positively associated with erectile function (Burns et al., 2010). A third study evaluated reports of men being treated for prostate cancer (Tsang et al., 2019). It found that the most important reason for men in this group to feel a diminished sense of their masculinity were their experience of the changes in their bodily functions, including the loss of the ability to achieve an erection, reduced libido, and impaired physical strength (Tsang et al., 2019). Many men with ED elect not to use ED medication, a practice which is influenced by men’s perception of ED as emasculating and of help-seeking as presenting a masculinity threat (Foster et al., 2022).

Overall, TMI appears to be weakly associated with ED severity (Wang, 2008; Komlenac & Hochleitner, 2022). In a study of 157 men living with ED, Wang (2008) found a weak association between ED severity and TMI. Wang also found an association between ED severity and the TMI subdimension “anti-femininity.” Corroborating these findings, in 261 self-identifying heterosexual male university students, anti-femininity endorsement was again positively correlated with erectile difficulties, while other TMI dimensions, including negativity toward sexual minorities, toughness, dominance, self-reliance, importance of sex, and restrictive emotionality, were not correlated with erectile difficulties (Komlenac & Hochleitner, 2022). Overall associations between TMI and ED were again found to be weak (Komlenac & Hochleitner, 2022).

However, no study has yet to investigate the relationship between male notions of manhood as elusive and ED. The recently developed Precarious Manhood Beliefs (PMB) scale (Bosson et al., 2021) provides a means through which to explore this relationship. PMB may be even more important to understanding and managing ED than TMI. Men may experience ED as the revocation of one’s manhood and a threat to one’s self-concept. Matters of causality are worth exploring. On the one hand, higher PMB may lead to greater pressure to perform sexually, which can reduce erectile function and promote ED (Liao et al., 2020; Vartolomei et al., 2022). On the other hand, developing ED may influence men’s psychology and promote PMB: ED may be experienced as a threat to these men's masculinity and their ability to identify as a satisfying sexual partner, leading men to ascribe to PMB.

The aim of the present study was to investigate the relationship between PMB and ED in cisgender men. Our stepwise analysis included a measure of conformity to TMI in order to disentangle the pure relationship between PMB and ED. TMI as well as measures of self-stigma, social desirability, and sociodemographic variables were controlled as covariates (Adegunloye & Ezeoke, 2011; Bergvall & Himelein, 2014; Fergus et al., 2002; Wiltink et al., 2003). The study explored four hypotheses:

- H1: In cisgender men, precarious manhood beliefs positively associate with symptoms of ED.
- H2: In cisgender men, precarious manhood beliefs positively associate with symptoms of ED when controlling for sociodemographic factors and social desirability.
- H3: In cisgender men, precarious manhood beliefs positively associate with symptoms of ED when controlling for sociodemographic factors, social desirability, and TMI.
- H4: In cisgender men, precarious manhood beliefs are positively associate with symptoms of ED when controlling for sociodemographic factors, social desirability, TMI, and self-stigma.

## Method

### Participants and Procedure

Data were derived from an anonymous online survey called Andromind Self-Test (AST). The survey collected data on sociodemographic information, mental and physical health, masculine gender role ideologies, and additional psychological constructs. Participant recruitment for the AST proceeded through geo-restricted advertisements on Facebook targeting adult men from German-speaking countries.

**Fig. 1:**
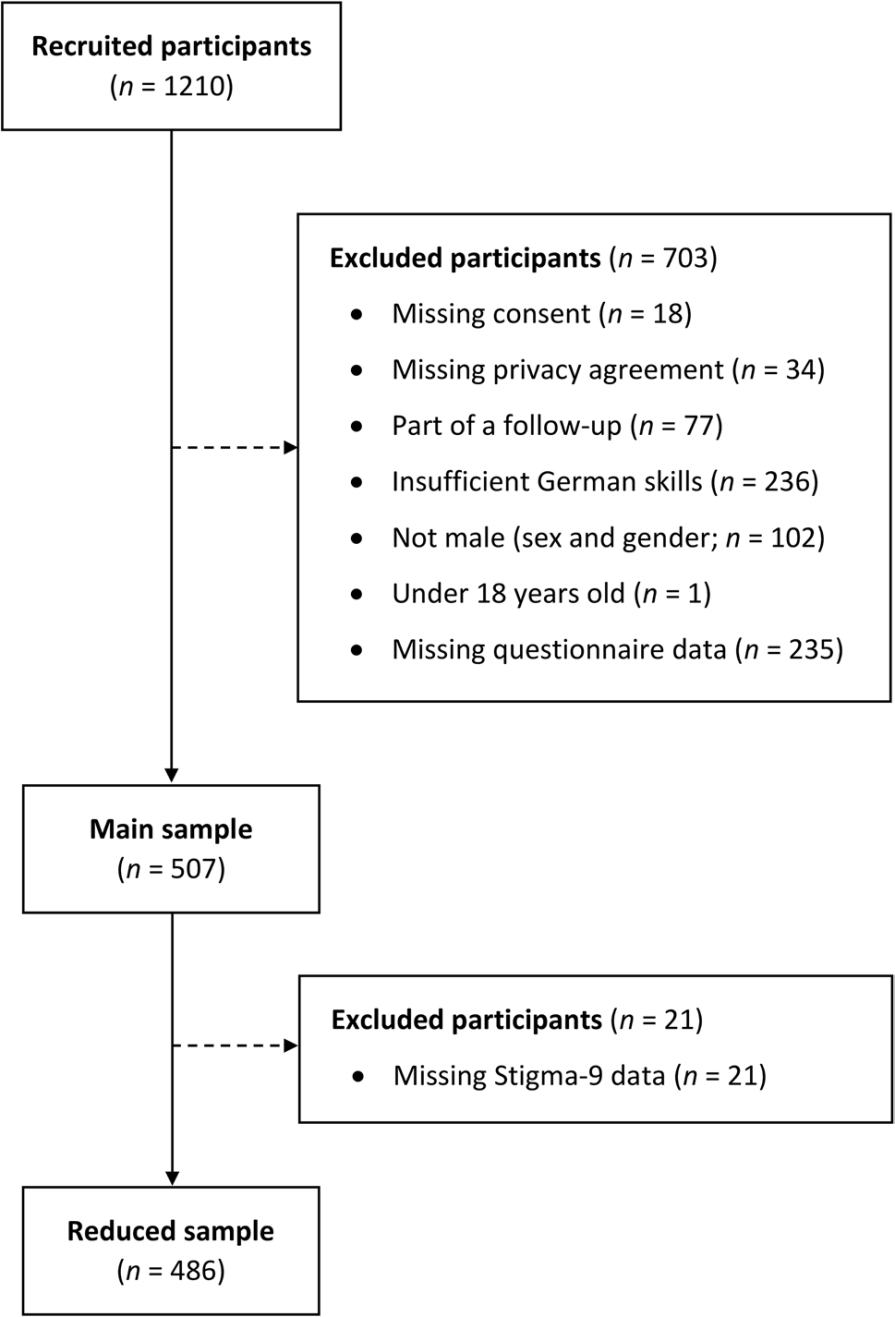
Overview of the exclusion process

To be eligible for participation, potential participants first had to consent to the privacy and data protection agreement and confirm that they were sufficiently proficient in the German language. Inclusion criteria included a participant age greater than 18 years, male sex assignment at birth, and current identification as a cisgender man. Participants had to complete all questionnaires relevant for the current study. This led to a main sample of 507 cisgender men. Because an additional questionnaire was used as a control variable in a few analyses, a second, slightly reduced sample of 486 cisgender men resulted for the respective estimations. A more detailed overview of the exclusion process is presented in Fig. 1.

### Measures

#### Sociodemographic Information

Participants provided sociodemographic information through responses to sequential questions about their gender identity, birth-assigned sex, age, nationality, yearly household income in Swiss Francs (CHF; conversion rate of 1 Euro to 1.11 CHF), highest educational attainment, self-identified sexual orientation, intimate and exclusive relationship status, and general health status. Furthermore, participants answered whether they were ever formally diagnosed with a psychiatric disorder as well as if they were currently taking any psychiatric medication. For the subsequent analyses, some of the answer options were aggregated into broader answer categories (e.g., heterosexual- and non-heterosexual-identified). A detailed overview of the questions and possible answer options, as well as the respective grouping for the analyses, can be found in the Supplementary (Table S1).

#### International Index of Erectile Function

The International Index of Erectile Function (IIEF; Rosen et al., 1997) is a well validated and widely used questionnaire assessing sexual function in men. Out of its 15 items, 12 measure erectile function (IIEF-EF; e.g., “How often were you able to get an erection during sexual activity?”), and three measure sexual desire (IIEF-SD; e.g., “How many times have you attempted sexual intercourse?”). For each item, participants indicated how often it occurred or how much it applied to them during the past four weeks on a five-point Likert scale (e.g., from 1 = *almost never/never* to 5 = *almost always/always*), including a null-option for most items such as 0 = *no sexual activity*. A score below 53 is used as cutoff for to indicate erectile dysfunction (Rosen et al., 1997). Furthermore, 10 supplementary items assessed additional aspects of sexual activity, for example intercourse frequency (“On average, how often do you have sexual intercourse?”) or use of medication for erectile dysfunction (“Have you ever taken Viagra or a similar drug in your life?”). Higher scores on the IIEF indicate a higher level of sexual functioning. The German language version used in the current study (Wiltink et al., 2003) showed excellent reliability for the total scale (Cronbach's α = 0.95) and the erectile function subscale (α = 0.96), but only fair reliability for the sexual desire subscale (α = 0.71). In the current sample, the IIEF showed good reliability for total scale (McDonald's ω = 0.95; α = 0.91) and the erectile function subscale (ω = 0.96; α = 0.92), but only fair to unsatisfactory reliability for the sexual desire subscale (ω = 0.73; α = 0.60).

#### Precarious Manhood Beliefs Scale

The Precarious Manhood Beliefs scale (PMB; Bosson et al., 2021) consists of four items assessing beliefs about whether manhood is difficult to earn (e.g., “Some boys do not become men no matter how old they get”) and whether it is easy to lose (e.g., “It is fairly easy for a man to lose his status as a man”). Participants indicate the extent to which they agree with each statement on a seven-point Likert scale, ranging from 1 (strongly disagree) to 7 (strongly agree). Higher scores on the PMB indicate a stronger belief that manhood is precarious. The back translated German version of the PMB has previously been found to possess unsatisfactory reliability in a large cross-cultural study among 1,864 people from Germany (ω = 0.69) and 581 people from Switzerland (ω = 0.66; Bosson et al., 2021). In the current sample, the PMB showed good reliability with ω = 0.85 and α = 0.81.

#### Stigma–9 Questionnaire

The Stigma–9 Questionnaire (STIG-9; Gierk et al., 2018) is a German questionnaire consisting of nine items that measure stigma associated with mental health. Participants indicated the extent to which they agree with a statement about negative societal beliefs, feelings, or behaviors regarding people with mental illnesses (e.g., “I think that most people consider mental illness to be a sign of personal weakness”). Higher scores on the STIG-9 indicate stronger stigmatization of mental illness. Possible answer options are presented on a four-point Likert scale ranging from 0 = *disagree* to 3 = *agree*. The STIG-9 showed good reliability in the original validation study with α = 0.88 (Gierk et al., 2018). In the present study, the STIG-9 showed excellent reliability with ω = 0.93 and α = 0.91.

#### Marlowe-Crowne Social Desirability Scale

The Marlowe-Crowne Social Desirability Scale (MC-SDS; Crowne & Marlowe, 1960) assesses social desirability (i.e., tendency of responding in a way thought to be expected by others) in participants’ response style. In the short-form used for the current study (Vésteinsdóttir et al., 2017), participants answered a series of 10 *yes–no* questions revolving around socially desirable traits or behaviors (e.g., “No matter who I talk to, I'm always a good listener”), for which a sum-score is then formed, with higher scores indicating a higher degree of social desirability. The original version of 10 items short-form showed good composite reliability with ρx = 0.83 (Vésteinsdóttir et al., 2017). The current study used a forward-translated German language version, which showed an unsatisfactory reliability in the present sample with ω = 0.67 and α = 0.61.

#### Conformity to Masculine Norms Inventory–30

The Conformity to Masculine Norms Inventory–30 (CMNI-30; Levant et al., 2020) is a short form of the CMNI (Mahalik et al., 2003) that consists of 30 items measuring conformity to TMI on the following 10 subscales: Emotional Control, Winning, Playboy, Violence, Heterosexual Self-Presentation (“Heterosexuality”), Pursuit of Status (“Status”), Primacy of Work (“Work”), Power over Women (“Patriarchic”), Self-Reliance, and Risk-Taking. Participants indicated how much they agree with different statements about TMI (e.g., “It bothers me when I have to ask for help.”) on a six-point Likert scale (0 = *strongly disagree* to 5 = *strongly agree*). Higher scores on the CMNI-30 indicate stronger conformity to TMI. The original English version of the CMNI-30 showed superior structural fit indices compared to alternative versions and fair (Status: α = 0.72) to excellent (Heterosexuality: α = 0.94) reliability (Levant et al., 2020). The current study used a back-translated German version of the CMNI-30 (Komlenac et al., 2023), which showed moderate overall reliability (ω = 0.88 and α = 0.85) in the present sample, ranging from unsatisfactory (Violence: ω = 0.67 and α = 0.67) to excellent (Heterosexuality: ω = 0.94 and α = 0.94) for the individual subscales.

### Statistical Analysis

The statistical analysis proceeded using an initial significance level of α = 0.05. This was subsequently adjusted for multiple hypothesis testing with a familywise Holm–Bonferroni correction (Holm, 1979) consisting of two main parts.

In the first part, descriptive statistics of the sociodemographic variables were calculated for men with and without clinically relevant ED (IIEF < 53). These groups were then compared by using Student’s* t*-test (applying the Welch-Satterthwaite approximation for heteroscedastic data; Welch, 1947) for continuous variables and Pearson’s chi-squared tests for categorical variables. Lastly, for all questionnaires used in the study, psychometric properties and Pearson’s bivariate correlation coefficients were estimated. The assessment of the questionnaires' reliability coefficients followed the model of Ponterotto and Ruckdeschel (2016).

In the second part, linear regression analyses were conducted with sexual function (IIEF total score), erectile function (IIEF-EF), and sexual desire (IIEF-SD) as individual outcomes with PMB as well as PMB and self-stigma (STIG-9) as predictor variables. A stepwise regression model was used to analyzed each of these six predictor–outcome combinations, including with (1) no covariates, (2) including as covariates participant’s age, income, education, and sexual orientation, and social desirability (MC-SDS), and (3) including as a covariate conformity to TMI (CMNI-30). Two post-hoc exploratory steps included (4) the CMNI-30 subscales that differed between the two groups (no ED vs. ED) or showed a significant correlation with the IIEF, and (5) a sensitivity analysis including covariates that differed significantly between the two groups (no ED vs. ED) but were initially not considered. Subsequent comparisons of nested models were conducted with *F*-tests. All regression coefficients were *z*-standardized to reduce multicollinearity and allow for direct a comparison of effects (Menard, 2011).

Normality of the studentized residuals could be assumed because of the large sample size (*n* > 500) as by the Central Limit Theorem. Furthermore, the residuals of all models were tested for dependencies using the Durbin-Watson test (Durbin & Watson, 1971; Fox, 2016; none present, all *p* > .05) and for heteroscedasticity using the Breusch-Pagan test (Breusch & Pagan, 1979; Fox, 2016). Because the Breusch-Pagan test indicated heteroscedastic residuals in one of the models (*p* < .05), robust standard error estimation was performed on that model (bias adjustment *HC3*; MacKinnon & White, 1985). Lastly, Cook’s distances (Cook, 1977) searched for highly influential points (none present, all *D* < 0.5) and the generalized variance inflation factor (Fox & Monette, 1992) assessed for potential multicollinearities among the predictor variables (none present, all *VIF* < 5).

All calculations were performed with the software *R* (version 4.1.2; R Core Team, 2020) and the additional *R*-packages *psych* (Revelle, 2020; estimation of bivariate correlations, psychometric properties, and implementation of the Holm–Bonferroni correction), *car* (Fox & Sanford, 2019; statistical tests for the assumption of regression models), and *sandwich* (Zeileis et al., 2020; estimation of robust standard errors).

## Results

### Descriptive Statistics and Group Comparisons

As presented in Table 1, participant ages ranged from 18 to 80 years old and averaged 44.2 years (*SD* = 15.2). The majority of men were German (69.6%), completed a secondary education (50.1%), self-identified as heterosexual (78.3%), and self-reported fair overall health (47.5%). About as many men were currently in an exclusive, intimate relationship (47.9%) as were not (45.4%). About one-third of the men in the sample self-reported to currently be diagnosed with a psychiatric disorder (30.6%), and about one-sixth were currently taking psychiatric medication (17.0%). Lastly, the majority of men showed clinically relevant signs of ED (63.1%) as indicated by an IIEF score below the cutoff of 53, 43% of men reported not having engaged in sexual intercourse during the past month, and about one-fifth had previously taken medication for ED (20.9%).

**Table 1:** Descriptive statistics stratified by erectile dysfunction (ED)

Variable	Total (n = 507)	No ED (n = 187)	ED (n = 320)	*t*/χ^2^ (df)	Effect size	*p*-value	*p* (corr.)
Age,* mean (SD)*	44.2 (15.2)	41.8 (14.3)	45.7 (15.5)	− 2.81 (505)	0.26	.005**	.010*
Nationality,* n (%)*				12.34 (4)	0.16	.015*	.060
Swiss	104 (20.5)	51 (27.3)	53 (16.6)				
German	353 (69.6)	114 (61.0)	239 (74.7)				
Austrian	36 (7.1)	15 (8.0)	21 (6.6)				
Luxembourger	1 (0.2)	1 (0.5)	0 (0.0)				
Other	5 (1.0)	2 (1.1)	3 (0.9)				
Yearly household income,* n (%)*				7.08 (2)	0.12	.029*	.087
< 25,000	153 (30.2)	50 (26.7)	103 (32.2)				
25,000–75,000	190 (37.5)	63 (33.7)	127 (39.7)				
> 50,000	164 (32.3)	74 (39.6)	90 (28.1)				
Education,* n (%)*				5.17 (3)	0.10	.160	.319
None completed	1 (0.2)	1 (0.5)	0 (0.0)				
Secondary education	254 (50.1)	84 (44.9)	170 (53.1)				
Tertiary education	224 (44.2)	89 (47.6)	135 (42.2)				
Sexual orientation,* n (%)*				4.77 (5)	0.10	.444	.444
Heterosexual	397 (78.3)	155 (82.9)	242 (75.6)				
Gay	60 (11.8)	16 (8.6)	44 (13.8)				
Bisexual	36 (7.1)	12 (6.4)	24 (7.5)				
Asexual	1 (0.2)	0 (0.0)	1 (0.3)				
Not sure/no answer	8 (1.6)	2 (1.1)	6 (1.9)				
Intimate Relationship,* n (%)*				134.48 (2)	0.52	** < **.001***	** < **.001***
Yes	243 (47.9)	149 (79.7)	94 (29.4)				
Yes, non-exclusive	34 (6.7)	15 (8.0)	19 (5.9)				
No	230 (45.4)	23 (12.3)	207 (64.7)				
General health,* n (%)*				26.35 (4)	0.23	** < **.001***	** < **.001***
Very bad	17 (3.4)	3 (1.6)	14 (4.4)				
Bad	115 (22.7)	25 (13.4)	90 (28.1)				
Fair	241 (47.5)	90 (48.1)	151 (47.2)				
Good	115 (22.7)	59 (31.6)	56 (17.5)				
Very good	19 (3.7)	10 (5.3)	9 (2.8)				
Psychiatric medication,* n (%)*	86 (17.0)	26 (13.9)	60 (18.8)	1.64 (1)	0.06	.200	.200
Psychiatric diagnosis,* n (%)*	155 (30.6)	34 (18.2)	121 (37.8)	20.51 (1)	0.21	** < **.001***	** < **.001**
Intercourse frequency*, n (%)*				280.44 (5)	0.74	** < **.001***	** < **.001***
Never	218 (43.0)	1 (0.5)	217 (67.8)				
Once a month	78 (15.4)	22 (11.8)	56 (17.5)				
2–3 times a month	70 (13.8)	51 (27.3)	19 (5.9)				
Once a week	71 (14.0)	57 (30.5)	14 (4.4)				
2–3 times a week	49 (9.7)	37 (19.8)	12 (3.8)				
> 3 times a week	21 (4.1)	19 (10.2)	2 (0.6)				
Viagra or similar medication, *n (%)*	106 (20.9)	36 (19.3)	70 (21.9)	0.35 (1)	0.03	.557	.557
Sexual function (IIEF)*, M (SD)*	42.8 (18.5)	64.1 (4.4)	30.3 (10.3)	51.16 (471)	− 3.93	** < **.001***	** < **.001***
Erectile function	34.4 (16.7)	53.6 (4.6)	23.1 (9.1)	49.78 (496)	− 3.92	** < **.001***	** < **.001***
Sexual desire	8.4 (3.0)	10.5 (1.9)	7.2 (2.8)	16.12 (495)	− 1.35	** < **.001***	** < **.001***
PMB,* M (SD)*	14.7 (6.2)	13.8 (6.5)	15.2 (6.1)	− 2.39 (505)	0.22	.017*	.069
STIG-9^a^,* M (SD)*	14.9 (5.8)	14.3 (5.8)	15.3 (5.7)	− 1.81 (484)	0.17	.070	.211
MC-SDS, *M (SD)*	4.3 (2.2)	4.2 (2.1)	4.4 (2.2)	− 0.77 (505)	0.07	.440	.879
CMNI-30,* M (SD)*	52.9 (17.4)	52.4 (17.3)	53.2 (17.5)	− 0.50 (505)	0.05	.615	.879
Emotional control	7.8 (3.8)	7.1 (3.7)	8.1 (3.8)	− 2.98 (505)	0.28	.003**	.030*
Winning	6.0 (3.2)	6.1 (3.4)	5.9 (3.1)	0.45 (505)	− 0.04	.652	.783
Playboy	5.5 (4.1)	6.0 (4.4)	5.2 (3.9)	1.96 (505)	− 0.18	.050	.410
Violence	4.6 (3.8)	4.9 (4.1)	4.4 (3.6)	1.47 (349)	− 0.14	.141	.424
Heterosexuality	3.9 (4.2)	3.4 (3.9)	4.1 (4.3)	− 1.86 (505)	0.17	.064	.410
Status	7.4 (3.2)	7.7 (3.1)	7.2 (3.2)	1.85 (505)	− 0.17	.065	.410
Work	5.4 (3.5)	5.0 (3.1)	5.6 (3.7)	− 1.97 (442)	0.17	.049*	.410
Patriarchic	2.2 (2.9)	2.1 (3.0)	2.3 (2.8)	− 0.86 (505)	0.08	.392	.783
Self-reliance	5.6 (3.6)	5.2 (3.5)	5.9 (3.6)	− 2.00 (505)	0.18	.046*	.410
Risk-taking	7.3 (3.6)	7.7 (3.6)	7.1 (3.7)	1.78 (505)	− 0.16	.075	.410

Bold values indicate statistical significance on an alpha-level of 5%

*n*, number of participants; *SD*, standard deviation; *Effect size*, Cohen’s d for continuous, Cramer’s V for categorical variables; *corr.*, adjusted for multiple testing using the Holm-method; *IIEF*, International Index of Erectile Function; *PMB*, Precarious Manhood Beliefs; *STIG-9*, Stigma-9 questionnaire; *MC-SDS*, Marlowe-Crowne Social Desirability Scale; *CMNI-30*, Conformity to Masculine Norms Inventory – 30

*p < .05; **p < .01; ***p < .001

^a^Calculations were performed with the reduced sample (*n* = 486)

As compared to men who did not fall below the cutoff for ED, men with ED were older, less often in an intimate relationship, in worse general health, more often diagnosed with a psychiatric disorder, and less often engaged in sexual intercourse. Furthermore, men with ED compared to men without ED had higher PMB but lower IIEF scores on erectile function and sexual desire subscales. The difference regarding PMB did not withstand a correction for multiple testing. Men with ED also reported stronger conformity to the TMI dimensions Emotional Control, Work, and Self-Reliance, though only the difference regarding Emotional Control persisted after correcting for multiple testing. No differences were found regarding self-stigma, social desirability, or overall conformity to TMI between the two groups.

### Correlation Analysis

As shown in Table 2, strong PMB correlated with lower sexual and erectile function, but not with sexual desire as well as with increased self-stigma, lower social desirability, and higher conformity to TMI. Sexual function, erectile function, and sexual desire were neither associated with self-stigma, social desirability, nor conformity to TMI. Strong conformity to TMI, on the other hand, correlated with increased self-stigma and lower social desirability. While conforming strongly to the TMI dimension Emotional Control was associated with lower sexual and erectile function, conforming strongly to the TMI dimension Playboy was linked to increased sexual desire.

**Table 2:** Pearson's correlation coefficients of the questionnaires

	1	1.1	1.2	2	3	4	5	5.1	5.2	5.3	5.4	5.5	5.6	5.7	5.8	5.9
1. Sexual function (IIEF)	–															
1.1 Erectile function	.99***	–														
1.2 Sexual desire	.66***	.56***	–													
2. PMB	− .16*	− .17**	− .02	–												
3. STIG-9^a^	− .11	− .12	.01	.29***	–											
4. MC-SDS	.00	.01	− .02	− .23***	− .14	–										
5. CMNI-30	− .05	− .07	.07	.42***	.19**	− .36***	–									
5.1 Emotional control	− .19**	− .19**	− .11	.26***	.10	− .04	.45***	–								
5.2 Winning	.00	.00	.01	.22***	.10	− .32***	.60	.11	–							
5.3 Playboy	.11	.08	.23***	.15	.09	− .25***	.47***	.02	.15*	–						
5.4 Violence	.05	.05	.05	.27***	.08	− .25***	.54***	.15	.28***	.20***	–					
5.5 Heterosexuality	− .11	− .13	.07	.29***	.14	− .16*	.52***	.17**	.25***	.08	.14	–				
5.6 Status	.11	.10	.10	.04	− .03	− .25***	.50	.04	.37***	.20***	.24***	.13	–			
5.7 Work	− .09	− .10	− .01	.14	.04	− .09	.46***	.15	.22***	.10	.09	.06	.24***	–		
5.8 Patriarchic	− .06	− .08	.04	.28***	.17*	− .19**	.58***	.19**	.33***	.23***	.25***	.37***	.22***	.15	–	
5.9 Self-reliance	− .12	− .12	− .09	.30	.16*	− .14	.50***	.34***	.20***	.11	.12	.20	.04	.11	.19***	–
5.10 Risk-taking	.08	.08	.07	.10	.09	− .16*	.40	− .03	.30	.18**	.20	.12	.16*	.22***	.07	.11

Bold values indicate statistical significance on an alpha-level of 5%

*p*-values were adjusted for multiple testing using the Holm-method

*IIEF*, International index of erectile function; *PMB*, Precarious manhood beliefs scale; *STIG-9*, Stigma-9 questionnaire; *MC-SDS*, Marlowe-Crowne Social Desirability Scale; *CMNI-30*, Conformity to Masculine Norms Inventory – 30

*p < .05; **p < .01; ***p < .001

^a^Calculations were performed with the reduced sample (*n* = 486)

### Multilinear Regression Analysis

Linear regression analyses examining the relationship between PMB and sexual function (Table 3) revealed that strong PMB were associated with reduced sexual function. This held even when controlling for sociodemographic covariates, social desirability, and conformity to overall TMI and corrected for multiple testing. After conducting a post-hoc sensitivity analysis by including relationship status, general health, psychiatric diagnosis, and intercourse frequency as covariates, the association between PMB and sexual function persisted but no longer withstood a correction for multiple testing. Strong PMB were also associated with reduced erectile function even when all covariates were included, but the association did not remain significant when the sensitivity analysis was corrected for multiple testing (Supplementary Table S3).

**Table 3:** Hierarchical linear regression Models 1–5 predicting sexual function (IIEF) with PMB

Predictor	Regression 1		Regression 2		Regression 3		Regression 4 (expl.)		Regression 5 (sens.)	
	β ^(adj.)^	95%-CI	β ^(adj.)^	95%-CI	β ^(adj.)^	95%-CI	β ^(adj.)^	95%-CI	β ^(adj.)^	95%-CI
PMB	− **0**.16***	**[**− **0**.**24, **− **0**.07]***	− **0**.15**	**[**− **0**.**24, **− **0**.06]***	− **0**.14*	**[**− **0**.**23, **− **0**.04]**	− 0.10	**[**− **0**.**20, **− **0**.01]*	− 0.07	**[**− **0**.**14, **− **0**.01]*
Age			− **0**.19***	**[**− **0**.**28, **− **0**.10]***	− **0**.19***	**[**− **0**.**28, **− **0**.10]***	− **0**.20***	**[**− **0**.**29, **− **0**.12]***	− **0**.14***	**[**− **0**.**20, **− **0**.07]***
Income			0.02	[− 0.07, 0.10]	0.02	[− 0.06, 0.11]	0.03	[− 0.06, 0.11]	− 0.02	[− 0.08, 0.04]
Education			**0**.30**	**[0**.**11, 0**.48]**	**0**.30**	**[0**.**12, 0**.48]**	0.25	**[0**.**07, 0**.43]**	0.16	**[0**.**04, 0**.28]*
Non-heterosexual			− 0.10	[− 0.31, 0.10]	− 0.11	[− 0.32, 0.10]	− 0.18	[− 0.38, 0.03]	− 0.10	[− 0.24, 0.04]
MC-SDS			− 0.01	[− 0.10, 0.08]	− 0.02	[− 0.11, 0.07]	0.02	[− 0.07, 0.11]	− 0.04	[− 0.10, 0.02]
CMNI-30					− 0.04	[− 0.14, 0.06]			− 0.04	[− 0.11, 0.03]
Emotional control							− **0**.15*	**[**− **0**.**24, **− **0**.06]**		
Playboy							**0**.14*	**[0**.**05, 0**.22]**		
Work							− 0.07	[− 0.16, 0.01]		
Self-reliance							− 0.06	[− 0.15, 0.03]		
Relationship									**0**.40***	**[0**.**26, 0**.53]***
General health									**0**.15***	**[0**.**09, 0**.21]***
Psych. diagnosis									− 0.12	[− 0.25, 0.01]
Freq. intercourse									**0**.55***	**[0**.**49, 0**.62]***
*Model fit*										
*F* (df_1_, df_2_)	12.79 (1, 505)		6.04 (6, 500)		5.26 (7, 499)		6.53 (10, 496)		64.88 (11, 495)	
BIC	4401.6		4410.0		4415.6		4407.7		4024.0	
Δ*R*^2^ in %	**2**.28***		**3**.37***		0.00		**4**.21***		**52**.56***	

Bold values indicate statistical significance on an alpha-level of 5%

*expl.*, post-hoc exploratory analyses; *sens.*, post-hoc sensitivity analyses; *β *^*(adj.)*^, standardized regression coefficient with significance level adjusted (adj.) for multiple testing using the holm method; *95%-CI*, two-sided 95% confidence interval with unadjusted significance level; *df*, degrees of freedom; *ΔR*^*2*^, change in adjusted *R*^2^ for nested consecutive (*R*^2^_1_; *R*^2^_2_—*R*^2^_1_; *R*^2^_3_—*R*^2^_2_; *R*^2^_4_—*R*^2^_2_; *R*^2^_5_—*R*^2^_3_) models; *IIEF*, International Index of Erectile Function; *PMB*, Precarious Manhood Beliefs scale; *STIG-9*, Stigma-9 questionnaire; *MC-SDS*, Marlowe-Crowne Social Desirability Scale; *CMNI-30*, Conformity to Masculine Norms Inventory – 30; *Psych.*, psychiatric; *Freq.*, frequency

*p < .05; **p < .01; ***p < .001

When self-stigma was used as an additional predictor, the associations between higher endorsement of PMB and reduced sexual function persisted (Table 4, Supplementary Table S5). So too did higher endorsement of PMB and reduced erectile function (Table 4, Supplementary Table S5). However, these effects did not withstand a correction for multiple testing nor a sensitivity analysis. Strong self-stigma was linked with reduced sexual function when controlling for sociodemographic covariates and social desirability (Table 4). This effect vanished after applying a Holm–Bonferroni correction as well as in the sensitivity analysis. Strong self-stigma was further associated with reduced erectile function when covariates were included and when conformity to TMI was considered (Supplementary Table S5), but the effect did not persist in the sensitivity analysis or with a correction for multiple testing. Lastly, PMB and self-stigma were not associated with sexual desire (Supplementary Tables S4 and S6).

**Table 4:** Hierarchical linear regression Models 1–5 predicting sexual function (IIEF) with PMB and STIG-9

Predictor	Regression 1		Regression 2		Regression 3		Regression 4 (expl.)		Regression 5 (sens.)	
	β ^(adj.)^	95%-CI	β ^(adj.)^	95%-CI	β ^(adj.)^	95%-CI	β ^(adj.)^	95%-CI	β ^(adj.)^	95%-CI
PMB	− **0**.13*	**[**− **0**.**22, **− **0**.03]**	− 0.12	**[**− **0**.**21, **− **0**.02]*	− 0.11	**[**− **0**.**21, **− **0**.01]*	− 0.08	[− 0.17, 0.02]	− 0.06	[− 0.13, 0.00]
STIG-9	− 0.07	[− 0.16, 0.02]	− 0.09	**[**− **0**.**18, 0**.00]*	− 0.09	[− 0.18, 0.00]	− 0.08	[− 0.17, 0.01]	− 0.03	[− 0.10, 0.03]
Age			− **0**.19***	**[**− **0**.**29, **− **0**.10]***	− **0**.20***	**[**− **0**.**29, **− **0**.11]***	− **0**.21***	**[**− **0**.**30, **− **0**.12]***	− **0**.14***	**[**− **0**.**21, **− **0**.08]***
Income			0.02	[− 0.07, 0.11]	0.02	[− 0.07, 0.11]	0.03	[− 0.06, 0.11]	− 0.02	[− 0.08, 0.04]
Education			**0**.32**	**[0**.**13, 0**.50]***	**0**.32**	**[0**.**14, 0**.51]***	**0**.26*	**[0**.**08, 0**.45]**	**0**.18*	**[0**.**05, 0**.30]**
non-heterosexual			− 0.05	[− 0.27, 0.16]	− 0.06	[− 0.27, 0.16]	− 0.13	[− 0.34, 0.08]	− 0.08	[− 0.23, 0.06]
MC-SDS			− 0.02	[− 0.11, 0.07]	− 0.03	[− 0.13, 0.06]	0.00	[− 0.09, 0.09]	− 0.05	[− 0.11, 0.02]
CMNI-30					− 0.04	[− 0.14, 0.07]			− 0.04	[− 0.11, 0.03]
Emotional control							− **0**.16**	**[**− **0**.**25, **− **0**.07]***		
Playboy							**0**.14*	**[0**.**05, 0**.23]**		
Work							− 0.08	[− 0.16, 0.01]		
Self-reliance							− 0.03	[− 0.13, 0.06]		
Relationship									**0**.40***	**[0**.**25, 0**.54]***
General health									**0**.14***	**[0**.**08, 0**.20]***
Psych. diagnosis									− 0.13	[− 0.26, 0.00]
Freq. intercourse									**0**.55***	**[0**.**48, 0**.62]***
Model fit										
*F* (df_1_, df_2_)	6.46 (2, 483)		5.39 (7, 478)		4.78 (8, 477)		6.23 (11, 474)		57.08 (12, 473)	
BIC	4228.9		4235.7		4241.4		4231.7		3868.5	
Δ*R*^2^ in %	**2**.20**		**3**.76***		0.00		**4**.64***		**52**.25***	

Bold values indicate statistical significance on an alpha-level of 5%

All calculations were performed with the reduced sample (*n* = 486);

*expl.*, post-hoc exploratory analyses; *sens.*, post-hoc sensitivity analyses; *β *^*(adj.)*^, standardized regression coefficient with significance level adjusted (adj.) for multiple testing using the holm method; *95%-CI*, two-sided 95% confidence interval with unadjusted significance level; *df*, degrees of freedom; *ΔR*^*2*^, change in adjusted *R*^2^ for nested consecutive (*R*^2^_1_; *R*^2^_2_—*R*^2^_1_; *R*^2^_3_—*R*^2^_2_; *R*^2^_4_—*R*^2^_2_; *R*^2^_5_—*R*^2^_3_) models; *IIEF*, International Index of Erectile Function; *PMB*, Precarious Manhood Beliefs scale; *STIG-9*, Stigma-9 questionnaire; *MC-SDS*, Marlowe-Crowne Social Desirability Scale; *CMNI-30*, Conformity to Masculine Norms Inventory – 30; *Psych.*, psychiatric; *Freq.*, frequency

*p < .05; **p < .01; ***p < .001

Because it was no longer evident from these models whether PMB or the TMI dimensions Emotional Control and Playboy were more strongly associated with men's sexual function, additional regression models were estimated to directly compare these effects under consideration of all covariates (Table 5). Therein, PMB showed a negative association with men's sexual function, while the Playboy dimension showed a positive association with men's sexual function, both being of comparable magnitude. The Emotional Control dimension had no significant association with men's sexual function. Importantly, the Playboy dimension primarily associated with men's sexual desire (Table 2; Table S4; Table S6).

**Table 5:** Linear regression models to compare the association of PMB, STIG-9, and relevant TMI subscales with sexual function (IIEF)

Predictor	Regression 1		Regression 2^a^	
	β ^(adj.)^	95%-CI	β ^(adj.)^	95%-CI
PMB	− 0.08	**[**− **0**.**15, **− **0**.02]**	− 0.08	**[**− **0**.**14, **− **0**.01]*
STIG-9			− 0.03	[− 0.10, 0.03]
Age	− **0**.14***	**[**− **0**.**20, **− **0**.07]***	− **0**.14***	**[**− **0**.**21, **− **0**.08]***
Income	− 0.02	[− 0.08, 0.04]	− 0.02	[− 0.08, 0.04]
Education	0.12	**[0**.**00, 0**.25]*	0.14	**[ 0**.**01, 0**.26]*
Non-heterosexual	− 0.12	[− 0.26, 0.02]	− 0.11	[− 0.26, 0.03]
MC-SDS	− 0.01	[− 0.07, 0.05]	− 0.02	[− 0.08, 0.04]
Emotional control	− 0.05	[− 0.11, 0.01]	− 0.06	[− 0.12, 0.00]
Playboy	0.08	**[0**.**02, 0**.14]**	0.08	**[0**.**02, 0**.15]**
Relationship	**0**.41***	**[0**.**27, 0**.55]***	**0**.41***	**[0**.**27, 0**.55]***
General health	**0**.14***	**[0**.**08, 0**.21]***	**0**.14***	**[0**.**07, 0**.20]***
Psych. diagnosis	− 0.12	[− 0.25, 0.01]	− 0.14	**[**− **0**.**27, 0**.00]*
Freq. intercourse	**0**.54***	**[0**.**47, 0**.61]***	**0**.53***	**[ 0**.**46, 0**.60]***
*Model fit*				
*F* (df_1_, df_2_)	60.93 (12, 494)		54.25 (13, 472)	
BIC	4022.4		3865.6	
*R*^2^ in %	**58**.7***		**58**.8***	

Bold values indicate statistical significance on an alpha-level of 5%

*expl.*, post-hoc exploratory analyses; *sens.*, post-hoc sensitivity analyses; *β *^*(adj.)*^, standardized regression coefficient with significance level adjusted (adj.) for multiple testing using the holm method; *95%-CI*, two-sided 95% confidence interval with unadjusted significance level; *df*, degrees of freedom; *R*^2^, adjusted *R*^2^; *IIEF*, International Index of Erectile Function; *PMB*, Precarious Manhood Beliefs scale; *STIG-9*, Stigma-9 questionnaire; *MC-SDS*, Marlowe-Crowne Social Desirability Scale; *CMNI-30*, Conformity to Masculine Norms Inventory – 30; *Psych.*, psychiatric; *Freq.*, frequency

*p < .05; **p < .01; ***p < .001

^a^Calculations were performed with the reduced sample (*n* = 486)

Model comparisons revealed that sociodemographic covariates consistently explained incremental variance on top of PMB and self-stigma in men’s sexual function and erectile function. The inclusion of conformity to TMI as a general factor did not improve the model fit, whereas including the (exploratorily determined) relevant TMI dimensions and the covariates for the sensitivity analyses improved the fit whereby men's relationship status and intercourse frequency seemed to explain the majority of variance.

## Discussion

### Summary of Results

This study is the first to establish a positive association between PMB and sexual function and erectile function. Moreover, the data suggest that men’s beliefs about the precariousness of manhood, namely PMB, associates with sexual dysfunction in men rather than their adherence to TMI. As all analyses were controlled for sociodemographic variables, social desirability, TMI, and self-stigma, the study demonstrated that stronger PMB associated with worse sexual function in cis-men even when accounting for relevant covariates. However, controlling for multiple testing resolved the association between PMB and sexual function, suggesting an overall small effect.

### Integration of Findings

This study highlights the importance of considering PMB in the etiology and treatment of sexual dysfunction, and of ED in particular, in men. Although the present study only demonstrates a cross-sectional positive association between PMB and sexual dysfunction in cisgender men, it underscores the relationship between these constructs. It supports the notion that PMB, rather than TMI, plays a critical role with regard to ED.

Previous studies have repeatedly shown that, except for the antifemininity subdimension, TMI are not associated with ED (Komlenac & Hochleitner, 2022; Thompson & Barnes, 2013; Wang, 2008). Strong conformity to TMI does not seem either to conflict with or reinforce ED because of the multiple possibilities of conforming to TMI (e.g., striving for status, being dominant, or being emotionally restrictive). Even if a man suffers from ED, he may, for example, remain dominant at work and strive for status, or be self-reliant and stoically carry the burden of ED. This is reflected in our extended analyses showing that of the two only relevant TMI subdimensions (Emotional Control and Playboy), only Playboy remained significantly associated with sexual function when considering PMB and covariates. This may be due to the positive relationship of Playboy to sexual desire but not erectile function (see Tables 2 and 5). Taken together, PMB remained a significant predictor of sexual function even when accounting for different TMI dimensions or the sensitivity analysis. However, due the increased number of predictors raising the α-level, as soon as three or more additional variables were introduced, the significant association between PMB and sexual function faded after correcting for multiple testing. This suggests that the effect of PMB on sexual function may be relatively small.

The previously established association between stronger endorsement of the TMI antifemininity subdimension and ED may reflect a compensatory response to stabilize a masculinity self-concept in the face of threat posed by ED (Eckes, 2008; Murnen et al., 2016; O’Neil et al., 1986). Supporting this hypothesis, experimentally induced masculinity threats prompt men to endorse stereotypically masculine attitudes and rate themselves as more masculine (Cheryan et al., 2015; Willer et al., 2013). Thus, when ED poses a masculinity threat, many men increase their motivation to see themselves as masculine and their masculine characteristics. Neither of these reactions alters their overall conformity to TMI (Dahl & Cook, 2020; Vandello & Bosson, 2013).

As a possible explanation for why PMB might be related to ED, one could speculate that men with strong PMB may put more pressure on themselves to perform sexually to allay anxieties. Men may worry that sexual failure related to ED may lead to the loss of their manhood. Men with strong PMB may believe that sexual performance is an integral part of normative masculinity (Fergus et al., 2002; Potts, 2000), and that the ability to get an erection reflects “manliness” itself (Baglia, 2005). Men with strong PMB may believe that penetrative sexual intercourse proves one’s masculinity and that ED threatens their manhood through a sense of inadequacy (McLaren, 2007). Men who do not have this conviction may therefore enter into sexual interactions in a more relaxed manner and may thus have a lower risk of erectile problems (McCabe, 2001).

If strong PMB and psychological pressure to perform sexually prevents an erection, the problem might perpetuate through losses of confidence (Andersson et al., 2011; Metz et al., 2017). This could create a vicious cycle of self-imposed pressure to perform sexually in order to maintain one’s manhood, increasing the risk for erectile problems. This may occur through overly high expectations and constant fear of losing one’s manhood, through self-inflicted rejection or via any confirmatory signals from the sexual partner, and through the conviction that one has now lost his manhood.

The conviction that manhood is not secure and that one can lose one's manhood plays a unique role in the area of masculine sexuality. No other facet stands so strongly for virility. The inability to achieve an erection may be conflated with not being able to achieve manhood. Thus, it is important that future treatment programs that use cognitive behavioral therapy to address ED in men (Andersson et al., 2011; McCabe, 2001; Metz et al., 2017), to explore and to clarify adherence to PMB in men, and, if necessary, to disconnect or even resolve PMB from ED. Similar attempts already exist with regard to the treatment of major depressive disorder in men (Seidler et al., 2022; Walther & Eggenberger, 2022; Walther et al., 2022) or psychotherapy uptake in general (Eggenberger et al., 2021, 2022). In these approaches, manhood can be detached from the ability to achieve an erection through disputation of PMB and cognitive restructuring. This may relieve the affected men of the double pressure of needing to achieve an erection and to stabilize the threatened masculinity self-concept.

It is noteworthy that self-stigma had a comparable effect on sexual function as did adherence to PMB with respect to the effect sizes found in the present sample. This finding contrasts with that of previous study among college students which suggested low levels of self-stigma in regard to potential sexual problems (Bergvall & Himelein, 2014). However, this study of college students consisted of a relatively small sample of mixed gender participants where cisgender men reported higher self-stigma in relation to potential sexual problems relative to the other groups, including cisgender women.

As self-stigma includes a person’s awareness of, agreement with, and internalization of stereotypes related to a mental disorder, the consequences of self-stigma can include diminished self-esteem, self-efficacy, and confidence in one’s future (Corrigan et al., 2010). Internalized ED stereotypes as identified in the Stereotypes About Male Sexuality Scale carry negative connotations with regard to one's manhood (e.g., “Lack of an erection will always spoil sex for a man” or “Men are almost always concerned with their sexual performance”) (Snell & Belk, 1990). Such widespread self-stigmatizing stereotypes overlap with PMB and thus may produce comparable effects. The positive association between self-stigma and PMB in the present study highlights the stigma of ED in regard to masculinity and the potential loss of manhood (Adegunloye & Ezeoke, 2011). This is consistent with the perspective that men who believe in the precariousness of manhood are also those who stigmatize themselves or other men when they behave unmanly or show effeminate characteristics such as ED.

Taken together, the fear of the potential loss of one's sexual function, as occurs in ED, may render men afraid of losing what makes them a man. ED may therefore represent a threat to masculinity and a potential loss of manhood status as rooted in the very concept of PMB. Although the expression of traits and behaviors to achieve and maintain manhood status differs across the ages, the underlying need to prove masculinity to obtain and to maintain manhood persists throughout history (Kimmel, 2018). The single feature that characterizes manhood is the persistent anxiety itself around achieving and maintaining manhood (Vandello & Bosson, 2013). It might be this anxiety around the maintenance and loss of manhood and the conflation of erectile function and penetrative intercourse with masculinity that increase the psychological stress of having to perform sexually. This in turn may negatively impact erectile function (Allen & Walter, 2019; Bocchio et al., 2009; Walther et al., 2017). This study sheds light on an emerging key construct, namely PMB, as a psychological cause and perpetuating factor of ED that may very well be treated by psychological means to reduce or even prevent the suffering of many men and their partners.

### Limitations

The present study must be interpreted with consideration of several limitations. The self-selection bias inherent in our methods limit generalizability. Because participation was based on interest in self-assessment of mental health status particularly designed for men, it is probable that a disproportionate number of men with mental distress participated in the survey. The high numbers of men who self-report suffering from a mental disorder and taking psychiatric medication supports this hypothesis. Since mental disorders are also often associated with sexual dysfunction, it is not surprising that such a high percentage (63%) of men in the current sample reach the cutoff point for clinically relevant signs of ED. Thus, the sample must be considered a sample with high psychological distress and high sexual dysfunction and cannot be considered representative of the general population of cisgender men.

The second important limitation is this study’s cross-sectional design. No conclusion can be drawn about cause and effect, since only associations could be investigated. Guided by theory, we hypothesized PMB as an underlying, early established, and enduring belief system and ED as a disorder that mostly emerges in adulthood. Therefore, PMB is used as a predictor and ED as an outcome variable in the multilinear regression models. But future longitudinal studies need to show that this assumed direction of association is justified and that it is not ED that provokes or reinforces PMB.

Finally, it can be argued that the sample studied is too small to include more relevant covariates in the models without creating power problems, which in turn renders associations non-significant when controlling for multiple testing.

### Conclusion

For the first time a positive association between PMB and ED in cisgender men was identified. The finding that PMB was consistently associated with ED must acknowledge that this association did not hold when corrected for multiple testing and showed a small effect size, and does not infer causality.

Nevertheless, the finding that it is not conformity to TMI but rather conformity to beliefs about the precariousness of manhood, namely PMB, that associates with sexual dysfunction in cisgender men carries important implications for the psychological treatment of sexual dysfunction. Mental health specialists may support men suffering from ED to eliminate the link between the notion that manhood can be lost and ED and reduce the pressure to perform sexually so that the masculine self-concept and a man’s self-worth are not affected by ED. In establishing new and diverse masculinities that are not threatened by a loss of erectile function (e.g., “I can also please my sexual partner without penetration” or “I am a romantic and passionate sexual partner irrespective of my ability to get an erection” or “Erectile function may come and go, but my manhood stays”), men can be helped to maintain mental health though experiencing ED. This, in turn, has a calming effect on sexual interactions so that men who suffer from ED can become increasingly sexually functional again in a pressure-free context and thereby regain erectile function.

### Supplementary Information

Below is the link to the electronic supplementary material.

Supplementary file1 (DOCX 54 KB)

## Data Availability

Following the Open Science standards, a priori defined study hypotheses and statistical analyses can be retrieved from OSF (https://osf.io/vt5s7). The data and code used for the present study will be made available by the corresponding author upon reasonable request.

## References

[CR1] Adegunloye OA, Ezeoke GG (2011). Sexual dysfunction—A silent hurt: Issues on treatment awareness. Journal of Sexual Medicine.

[CR2] Allen MS, Walter EE (2019). Erectile dysfunction: An umbrella review of meta-analyses of risk-factors, treatment, and prevalence outcomes. Journal of Sexual Medicine.

[CR3] Andersson E, Walén C, Hallberg J, Paxling B, Dahlin M, Almlöv J, Källström R, Wijma K, Carlbring P, Andersson G (2011). A randomized controlled trial of guided internet-delivered cognitive behavioral therapy for erectile dysfunction. Journal of Sexual Medicine.

[CR100] Baglia J (2005). The Viagra ad venture: Masculinity, marketing, and the performance of sexual health.

[CR4] Bergvall L, Himelein MJ (2014). Attitudes toward seeking help for sexual dysfunctions among US and Swedish college students. Sexual and Relationship Therapy.

[CR5] Bocchio M, Pelliccione F, Mihalca R, Ciociola F, Necozione S, Rossi A, Francavilla F, Francavilla S (2009). Treatment of erectile dysfunction reduces psychological distress. International Journal of Andrology.

[CR6] Bosson JK, Jurek P, Vandello JA, Kosakowska-Berezecka N, Olech M, Besta T, Bender M, Hoorens V, Becker M, Timur Sevincer A, Best DL, Safdar S, Włodarczyk A, Zawisza M, Żadkowska M, Abuhamdeh S, Badu Agyemang C, Akbaş G, Albayrak-Aydemir N, Žukauskienė R (2021). Psychometric properties and correlates of precarious manhood beliefs in 62 nations. Journal of Cross-Cultural Psychology.

[CR7] Breusch TS, Pagan AR (1979). A simple test for heteroscedasticity and random coefficient variation. Econometrica.

[CR8] Burkley M, Wong YJ, Bell AC (2016). The Masculinity contingency scale (MCS): Scale development and psychometric properties. Psychology of Men & Masculinity.

[CR9] Burns SM, Hough S, Boyd BL, Hill J (2010). Men’s adjustment to spinal cord injury: The unique contributions of conformity to masculine gender norms. American Journal of Men’s Health.

[CR10] Cheryan S, Schwartz Cameron J, Katagiri Z, Monin B (2015). Manning up: Threatened men compensate by disavowing feminine preferences and embracing masculine attributes. Social Psychology.

[CR11] Cook RD (1977). Detection of influential observation in linear regression. Technometrics.

[CR12] Corrigan PW, Larson JE, Kuwabara SA, Maddux JE, Tangney JP (2010). Social psychology of the stigma of mental illness: Public and self-stigma models. Social psychological foundations of clinical psychology.

[CR13] Crowne DP, Marlowe D (1960). A new scale of social desirability independent of psychopathology. Journal of Consulting Psychology.

[CR14] Dahl, J., & Cook, J. (2020). *Masculinity may be an important, unstable self-concept that men psychologically defend*. Unpublished manuscript (Preprint).

[CR15] Durbin J, Watson GS (1971). Testing for serial correlation in least squares regression. III. Biometrika.

[CR16] Eckes, T. (2008). Geschlechterstereotype: Von Rollen, Identitäten und Vorurteilen. In R. Becker & B. Kortendiek (Eds.), *Handbuch Frauen- und Geschlechterforschung*. VS Verlag für Sozialwissenschaften. 10.1007/978-3-531-91972-0_20

[CR17] Eggenberger L, Fordschmid C, Ludwig C, Weber S, Grub J, Komlenac N, Walther A (2021). Men’s psychotherapy use, male role norms, and male-typical depression symptoms: Examining 716 men and women experiencing psychological distress. Behavioral Sciences.

[CR18] Eggenberger L, Komlenac N, Ehlert U, Grub J, Walther A (2022). Association between psychotherapy use, sexual orientation, and traditional masculinity among psychologically distressed men. Psychology of Men & Masculinities.

[CR19] Fergus KD, Gray RE, Fitch MI (2002). Sexual dysfunction and the preservation of manhood: Experiences of men with prostate cancer. Journal of Health Psychology.

[CR20] Foster S, Pomerantz A, Bell K, Carvallo M, Lee J, Lee J (2022). Victims of virility: Honor endorsement, stigma, and men’s use of erectile dysfunction medication. Psychology of Men and Masculinity.

[CR21] Fox J, Sanford W (2019). An R companion to applied regression.

[CR22] Fox J (2016). Applied regression analysis and generalized linear models.

[CR23] Fox J, Monette G (1992). Generalized collinearity diagnostics. Journal of the American Statistical Association.

[CR24] Gerdes ZT, Alto KM, Jadaszewski S, D’Auria F, Levant RF (2018). A content analysis of research on masculinity ideologies using all forms of the Male Role Norms Inventory (MRNI). Psychology of Men & Masculinity.

[CR25] Gierk B, Löwe B, Murray AM, Kohlmann S (2018). Assessment of perceived mental health-related stigma: The Stigma-9 Questionnaire (STIG-9). Psychiatry Research.

[CR26] Holm S (1979). A simple sequentially rejective multiple test procedure. Scandinavian Journal of Statistics.

[CR27] Kessler A, Sollie S, Challacombe B, Briggs K, van Hemelrijck M (2019). The global prevalence of erectile dysfunction: A review. BJU International.

[CR102] Kimmel MS, Brod H (2018). The contemporary “crisis” of masculinity in historical perspective. The making of masculinities.

[CR201] Komlenac N, Eggenberger L, Walther A, Maresch F, Lamp E, Hochleitner M (2023). Measurement invariance and psychometric properties of a German-language Conformity to Masculine Norms Inventory among cisgender sexual minority and heterosexually identified women and men. Psychology of Men & Masculinities.

[CR28] Komlenac N, Hochleitner M (2022). Heterosexual-identified men’s endorsement of masculinity ideologies moderates associations between pornography consumption, body satisfaction and sexual functioning. Psychology and Sexuality.

[CR29] Levant RF, Richmond K, Wong YJ, Wester SR (2016). The gender role strain paradigm and masculinity ideologies. APA handbook of men and masculinities.

[CR30] Levant RF, McDermott R, Parent MC, Alshabani N, Mahalik JR, Hammer JH (2020). Development and evaluation of a new short form of the Conformity to Masculine Norms Inventory (CMNI-30). Journal of Counseling Psychology.

[CR31] Levy GD, Taylor MG, Gelman SA (1995). Traditional and evaluative aspects of flexibility in gender roles, social conventions, moral rules, and physical laws. Child Development.

[CR32] Liao Z, Li X, Tang Y, Li D, Tang Z (2020). Is milder psychological stress responsible for more severe erectile dysfunction?. Andrologia.

[CR33] Loe M (2001). Fixing broken masculinity: Viagra asa technology for the production of gender and sexuality. Sexuality and Culture.

[CR34] MacKinnon JG, White H (1985). Some heteroskedasticity-consistent covariance matrix estimators with improved finite sample properties. Journal of Econometrics.

[CR35] Mahalik JR, Locke BD, Ludlow LH, Diemer MA, Scott RPJ, Gottfried M, Freitas G (2003). Development of the Conformity to Masculine Norms Inventory. Psychology of Men & Masculinity.

[CR36] McCabe MP (2001). Evaluation of a cognitive behavior therapy program for people with sexual dysfunction. Journal of Sex & Marital Therapy.

[CR37] McCreary DR (1994). The male role and avoiding femininity. Sex Roles.

[CR101] McLaren A (2007). The biggest flop of all. New Scientist.

[CR38] McVary KT (2007). Erectile dysfunction. New England Journal of Medicine.

[CR39] Menard S (2011). Standards for standardized logistic regression coefficients. Social Forces.

[CR40] Metz ME, Epstein NB, McCarthy B (2017). Cognitive-behavioral therapy for sexual dysfunction.

[CR41] Murnen SK, Greenfield C, Younger A, Boyd H (2016). Boys act and girls appear: A content analysis of gender stereotypes associated with characters in children’s popular culture. Sex Roles.

[CR42] O’Neil JM (2013). Gender role conflict research 30 years later: An evidence-based diagnostic schema to assess boys and men in counseling. Journal of Counseling & Development.

[CR43] O’Neil JM, Helms BJ, Gable RK, David L, Wrightsman LS (1986). Gender-Role Conflict Scale: College men’s fear of femininity. Sex Roles.

[CR44] Ponterotto JG, Ruckdeschel DE (2016). An overview of coefficient alpha and a reliability matrix for estimating adequacy of internal consistency coefficients with psychological research measures. Perceptual and Motor Skills.

[CR45] Potts A (2000). The essence of the hard on hegemonic masculinity and the cultural construction of erectile dysfunction. Men and Masculinities.

[CR46] R Core Team. (2020). *R: A language and environment for statistical computing* (4.0.3). R Foundation for Statistical Computing, Vienna, Austria.

[CR47] Revelle, W. (2020). *psych: Procedures for personality and psychological research* (2.1.3). Northwestern University, Evanston, Illinois, USA.

[CR48] Rosen RC, Riley A, Wagner G, Osterloh IH, Kirkpatrick J, Mishra A (1997). The International Index of Erectile Function (IIEF): A multidimensional scale for assessment of erectile dysfunction. Urology.

[CR49] Sand MS, Fisher W, Rosen R, Heiman J, Eardley I (2008). Erectile dysfunction and constructs of masculinity and quality of life in the Multinational Men’s Attitudes to Life Events and Sexuality (MALES) Study. Journal of Sexual Medicine.

[CR50] Seidler, Z. E., Wilson, M. J., Toogood, N. W., Oliffe, J. L., Kealy, D., Ogrodniczuk, J. S., Owen, J., Mackinnon, A., Le, L.K.-D., & Mihalopoulos, C. (2022). Protocol for a randomized controlled trial of the Men in Mind training for mental health practitioners to enhance their clinical competencies for working with male clients. *BMC Psychology,**10*(1). 10.1186/s40359-022-00875-9 10.1186/s40359-022-00875-9 PMC9288021 35841082

[CR51] Sirin SR, McCreary DR, Mahalik JR (2004). Differential reactions to men and women’s gender role transgressions: Perceptions of social status, sexual orientation, and value dissimilarity. Journal of Men’s Studies.

[CR52] Snell WE, Belk SS, Hawkins RC, Beere CA (1990). Stereotypes about male sexuality scale (SAMSS). Gender roles: A handbook of tests and measures.

[CR53] Thompson EH, Barnes K (2013). Meaning of sexual performance among men with and without erectile dysfunction. Psychology of Men & Masculinity.

[CR54] Tsang VWL, Skead C, Wassersug RJ, Palmer-Hague JL (2019). Impact of prostate cancer treatments on men’s understanding of their masculinity. Psychology of Men and Masculinity.

[CR55] Ussher JM, Perz J, Rose D, Dowsett GW, Chambers S, Williams S, Davis I, Latini D (2017). Threat of sexual disqualification: The consequences of erectile dysfunction and other sexual changes for gay and bisexual men with prostate cancer. Archives of Sexual Behavior.

[CR56] Vandello JA, Bosson JK (2013). Hard won and easily lost: A review and synthesis of theory and research on precarious manhood. Psychology of Men and Masculinity.

[CR57] Vandello JA, Bosson JK, Cohen D, Burnaford RM, Weaver JR (2008). Precarious manhood. Journal of Personality and Social Psychology.

[CR58] Vartolomei L, Cotruș A, Tătaru SO, Vartolomei MD, Man A, Ferro M, Stanciu C, Sin AI, Shariat SF (2022). Lower urinary tract symptoms are associated with clinically relevant depression, anxiety, and stress symptoms. The Aging Male.

[CR59] Vésteinsdóttir V, Reips UD, Joinson A, Thorsdottir F (2017). An item level evaluation of the Marlowe–Crowne Social Desirability Scale using item response theory on Icelandic Internet panel data and cognitive interviews. Personality and Individual Differences.

[CR61] Walther, A., & Eggenberger, L. (2022). Evaluation of male-specific psychoeducation for major depressive disorder compared to cognitive behavioral therapy psychoeducation: A randomized controlled investigation in mentally distressed men. *PsyArxiv*. 10.31234/osf.io/ru9ca 10.1080/10503307.2024.2398085 39257054

[CR60] Walther, A., Ehlert, U., Schneeberger, M., Eggenberger, L., Flückiger, C., Komlenac, N., Heald, A., Rice, T., Palm, S., & Seidler, Z. (2022). Evaluation of a male-specific psychotherapeutic program for major depressive disorder compared to cognitive behavioral therapy and waitlist: Study protocol for a six-arm randomized clinical superiority trial examining depressed eugonadal and hypogonadal men receiving testosterone. *PsyArxiv*. 10.31234/osf.io/7k2q4 10.3389/fpsyt.2023.1129386 PMC10321526 37415687

[CR62] Walther A, Mahler F, Debelak R, Ehlert U (2017). Psychobiological protective factors modifying the association between age and sexual health in men: Findings from the Men’s Health 40+ study. American Journal of Men’s Health.

[CR63] Wang, P. S. C. (2008). *The relationship between erectile function, traditional masculine ideology, and marital satisfaction among younger and older Taiwanese men with erectile dysfunction*. Alliant International University, Los Angeles. https://www.proquest.com/dissertations-theses/relationship-between-erectile-function/docview/304822208/se-2

[CR64] Welch ABL (1947). The generalization of student’s problem when several different population variances are involved. Biometrika.

[CR65] Willer R, Rogalin CL, Conlon B, Wojnowicz MT (2013). Overdoing gender: A test of the masculine overcompensation thesis. American Journal of Sociology.

[CR66] Wiltink J, Hauck EW, Phädayanon M, Weidner W, Beutel ME (2003). Validation of the German version of the International Index of Erectile Function (IIEF) in patients with erectile dysfunction, Peyronie’s disease and controls. International Journal of Impotence Research.

[CR67] Wong YJ, Burkley M, Bell AC, Wang S-Y, Klann EM (2017). Manly to the core: Measuring men’s implicit masculine self-concept via the semantic misattribution procedure. Personality and Individual Differences.

[CR68] Zaider T, Manne S, Nelson C, Mulhall J, Kissane D (2012). Loss of masculine identity, marital affection, and sexual bother in men with localized prostate cancer. Journal of Sexual Medicine.

[CR69] Zeileis A, Köll S, Graham N (2020). Various versatile variances: An object-oriented implementation of clustered covariances in R. Journal of Statistical Software.

