# Supplementary material for: Precarious Manhood Beliefs Are Positively Associated with Erectile Dysfunction in Cisgender Men
Source: Arch Sex Behav. 2023 Jun 23;52(7):3123–38. doi: 10.1007/s10508-023-02640-4 (PMC10684399; doi:10.1007/s10508-023-02640-4)
Supplement: Supplementary file 1 — Supplementary file1 (DOCX 54 KB) [file 10508_2023_2640_MOESM1_ESM.docx]

**Precarious Manhood Beliefs are Positively Associated With Erectile Dysfunction in Cisgender Men**

**Supplementary Materials**

[blinded]

**Content**

| **Table S1.** | *Overview of Sociodemographic Questions, Answer Options, Groupings, and Reference Levels* | page 2 |
| --- | --- | --- |
| **Table S2.** | *Psychometric Properties of the Questionnaires and Item-Level Mean Scores* | page 3 |
| **Table S3.** | *Hierarchical Linear Regression Models 1-5 Predicting Erectile Function (IIEF-EF) with PMB* | page 4 |
| **Table S4.** | *Hierarchical Linear Regression Models 1-5 Predicting Sexual Desire (IIEF-SD) with PMB* | page 5 |
| **Table S5.** | *Hierarchical Linear Regression Models 1-5 Predicting Erectile Function (IIEF-EF) with PMB and STIG-9* | page 6 |
| **Table S6.** | *Hierarchical Linear Regression Models 1-5 Predicting Sexual Desire (IIEF-SD) with PMB and STIG-9* | page 7 |

| **Table S1** *Overview of Sociodemographic Questions, Answer Options, Groupings, and Reference Levels* | |
| --- | --- |
| Q*:* | “What is your current gender identity?” |
| A: | 1 = *male*; 2 = *female*; 3 = *female-to-male transgender*; 4 = *male-to-female-transgender;* 5 = *gender neutral / non-binary;* 6 = *diverse*; 7 = *other* (with a free-text option); 8 = *don’t know / not sure* |
| Q: | “What sex were you assigned at birth ("biological sex")?” |
| A: | 1 = *male*; 2 = *female*; 3 = *diverse*; 4 = *other* (with a free-text option); 5 = *don’t know / not sure* |
| Q: | “Please enter your age in years (e.g., 42).” |
| A: | free-text option |
| Q: | “Which nationality do you belong to?” |
| A: | 1 = *Swiss*; 2 = *German*; 3 = *Austrian*; 4 = *Liechtensteiner*; 5 = *Luxembourger*;  6 = *Belgian*; 7 = *other* |
| Q: | “How much is the annual gross income of your household (in CHF)? (Note: 1 Euro = 1.11 CHF)” |
| A: | free-text option |
| Q: | “What is your highest completed education?” |
| A: | 1 = *no completed education*; 2 = *special school*; 3 = *secondary school*;  4 = *high school* ("Realschule"); 5 = *basic apprenticeship*; 6 = *apprenticeship*;  7 = *gymnasium* ("Abitur"); 8 = *university*; 9 = *other* |
| Grp.: | 1 = *none completed*; 2 = *secondary education*; 3 = *tertiary education*; 4 = *other* |
| Ref.: | 0 = *no tertiary education* |
| Q: | “What is your sexual orientation?” |
| A: | 1 = *heterosexual*; 2 = *gay / lesbian*; 3 = *bisexual*; 4 = *asexual*; 5 = *other* (with a free-text option); 6 = *don’t know / not sure* |
| Ref.: | 0 = *heterosexual* |
| Q: | “Are you currently in an intimate relationship?” |
| A: | 1 = *yes*; 2 = *no*; 3 = *yes, but not in an exclusive relationship* |
| Ref.: | 0 = *no intimate relationship* |
| Q: | “How would you rate your health in general?” |
| A: | 1 = *very bad*; 2 = *bad*; 3 = *fair*; 4 = *good*; 5 = *very good* |
| Q: | “Are you currently taking any psychiatric medications?” |
| A: | 1 = *yes*; 2 = *no* |
| Ref.: | 0 = *no psychiatric medication* |
| Q: | “Are you suffering from a diagnosed acute or chronic psychiatric disorder?” |
| A: | 1 = *yes*; 2 = *no* |
| Ref.: | 0 = *no psychiatric disorder* |
| *Note*. Q = translated question; A = translated answer options; Grp. = groupings used for sample description; Ref. = reference level used for the analyses (dummy-coded) | |

| **Table S2** *Psychometric Properties of the Questionnaires and Item-Level Mean Scores* | | | | | | | |
| --- | --- | --- | --- | --- | --- | --- | --- |
| Questionnaire | *n*_items_ | α | ω | mean (SD) | range | skewness | kurtosis |
| **Sexual function (IIEF)** | 15 | .91 | .95 | 2.85 (1.23) | [0; 5] | 0.00 | -1.22 |
| Erectile function | 12 | .92 | .96 | 2.64 (1.28) |  | 0.03 | -1.19 |
| Sexual desire | 3 | .60 | .73 | 2.80 (0.99) |  | -0.09 | -0.58 |
| **PMB** | 4 | .81 | .85 | 3.68 (1.56) | [1; 7] | 0.06 | -0.73 |
| **STIG-9 ^a^** | 9 | .91 | .93 | 1.66 (0.64) | [0; 3] | -0.35 | 0.02 |
| **MC-SDS** | 10 | .61 | .67 | 0.43 (0.22) | [0; 1] | 0.11 | -0.60 |
| **CMNI-30** | 30 | .85 | .88 | 1.76 (0.58) | [0; 5] | 0.67 | 1.26 |
| Emotional Control | 3 | .92 | .92 | 2.58 (1.26) |  | 0.10 | -0.74 |
| Winning | 3 | .77 | .80 | 1.99 (1.07) |  | 0.27 | -0.14 |
| Playboy | 3 | .83 | .85 | 1.84 (1.37) |  | 0.48 | -0.59 |
| Violence | 3 | .67 | .67 | 1.53 (1.27) |  | 0.60 | -0.38 |
| Heterosexuality | 3 | .94 | .94 | 1.29 (1.39) |  | 1.03 | 0.25 |
| Status | 3 | .72 | .73 | 2.47 (1.06) |  | 0.03 | -0.24 |
| Work | 3 | .83 | .84 | 1.80 (1.17) |  | 0.38 | -0.37 |
| Patriarchic | 3 | .82 | .83 | 0.75 (0.96) |  | 1.62 | 2.83 |
| Self-Reliance | 3 | .71 | .73 | 1.88 (1.19) |  | 0.14 | -0.82 |
| Risk-Taking | 3 | .90 | .90 | 2.44 (1.22) |  | 0.20 | -0.51 |
| *Note.* *n*_items_ = number of items; α = Cronbach’s alpha; ω = McDonald’s omega;  IIEF = International Index of Erectile Function; PMB = Precarious Manhood Beliefs scale; STIG-9 = Stigma-9 Questionnaire; MC-SDS = Marlowe-Crowne Social Desirability Scale; CMNI-30 = Conformity to Masculine Norms Inventory – 30. ^a^ Calculations were performed with the reduced sample (*n* = 486) | | | | | | | |

| **Table S3**  *Hierarchical Linear Regression Models 1-5 Predicting Erectile Function (IIEF-EF) with PMB* | | | | | | | | | | |
| --- | --- | --- | --- | --- | --- | --- | --- | --- | --- | --- |
| **Predictor** | **Regression 1** | | **Regression 2** | | **Regression 3** | | **Regression 4 (expl.)** | | **Regression 5 (sens.)** | |
|  | β ^(adj.)^ | 95%-CI | β ^(adj.)^ | 95%-CI | β ^(adj.)^ | 95%-CI | β ^(adj.)^ | 95%-CI | β ^(adj.)^ | 95%-CI |
| PMB | **-0.17^***^** | **[-0.26, -0.09]^***^** | **-0.17^***^** | **[-0.26, -0.08]^***^** | **-0.15^*^** | **[-0.24, -0.05]^**^** | -0.12 | **[-0.21, -0.03]^*^** | -0.08 | **[-0.14, -0.02]^*^** |
| Age |  |  | **-0.20^***^** | **[-0.29, -0.11]^***^** | **-0.21^***^** | **[-0.30, -0.12]^***^** | **-0.22^***^** | **[-0.30, -0.13]^***^** | **-0.15^***^** | **[-0.22, -0.09]^***^** |
| Income |  |  | 0.02 | [-0.07, 0.10] | 0.02 | [-0.06, 0.11] | 0.03 | [-0.06, 0.11] | -0.02 | [-0.07, 0.04] |
| Education |  |  | **0.27^*^** | **[0.09, 0.45]^**^** | **0.28^*^** | **[0.10, 0.46]^**^** | 0.24 | **[0.06, 0.42]^**^** | 0.14 | **[0.02, 0.26]^*^** |
| Non-Heterosexual |  |  | -0.12 | [-0.33, 0.08] | -0.13 | [-0.34, 0.07] | -0.19 | [-0.39, 0.02] | -0.12 | [-0.26, 0.02] |
| MC-SDS |  |  | -0.01 | [-0.09, 0.08] | -0.02 | [-0.11, 0.07] | 0.01 | [-0.07, 0.10] | -0.05 | [-0.11, 0.01] |
| CMNI-30 |  |  |  |  | -0.06 | [-0.16, 0.04] |  |  | -0.06 | [-0.13, 0.01] |
| Emotional Control |  |  |  |  |  |  | **-0.15^*^** | **[-0.24, -0.06]^**^** |  |  |
| Playboy |  |  |  |  |  |  | 0.11 | **[0.02, 0.20]^*^** |  |  |
| Work |  |  |  |  |  |  | -0.08 | [-0.16, 0.01] |  |  |
| Self-Reliance |  |  |  |  |  |  | -0.05 | [-0.14, 0.04] |  |  |
| Relationship |  |  |  |  |  |  |  |  | **0.41^***^** | **[0.28, 0.55]^***^** |
| General Health |  |  |  |  |  |  |  |  | **0.15^***^** | **[0.09, 0.21]^***^** |
| Psych. Diagnosis |  |  |  |  |  |  |  |  | -0.13 | [-0.26, 0.00] |
| Freq. Intercourse |  |  |  |  |  |  |  |  | **0.55^***^** | **[0.49, 0.62]^***^** |
| **Model Fit** |  |  |  |  |  |  |  |  |  |  |
| *F* (df_1_, df_2_) | 15.34 (1, 505) | | 6.75 (6, 500) | | 5.98 (7, 499) | | 6.63 (10, 496) | | 69.21 (11, 495) | |
| BIC | 4293.7 | | 4300.6 | | 4305.4 | | 4301.4 | | 3899.0 | |
| Δ*R*^2^ in % | **2.76^***^** | | **3.63^***^** | | 0.06 | | **3.63^***^** | | **53.28^***^** | |
| *Note.* expl. = post-hoc exploratory analyses; sens. = post-hoc sensitivity analyses; β ^(adj.)^ = standardized regression coefficient with significance level adjusted (adj.) for multiple testing using the holm method; 95%-CI = two-sided 95% confidence interval with unadjusted significance level; df = degrees of freedom; Δ*R*^2^ = change in adjusted *R*^2^ for nested consecutive (*R*^2^_1_; *R*^2^_2_ - *R*^2^_1_; *R*^2^_3_ - *R*^2^_2_; *R*^2^_4_ - *R*^2^_2_; *R*^2^_5_ - *R*^2^_3_) models; IIEF = International Index of Erectile Function; PMB = Precarious Manhood Beliefs scale; STIG-9 = Stigma-9 questionnaire; MC-SDS = Marlowe-Crowne Social Desirability Scale; CMNI-30 = Conformity to Masculine Norms Inventory – 30; Psych. = psychiatric; Freq. = frequency. ^*^ *p* < .05; ^**^ *p* < .01; ^***^ *p* < .001 | | | | | | | | | | |

| **Table S4**  *Hierarchical Linear Regression Models 1-5 Predicting Sexual Desire (IIEF-SD) with PMB* | | | | | | | | | | |
| --- | --- | --- | --- | --- | --- | --- | --- | --- | --- | --- |
| **Predictor** | **Regression 1** | | **Regression 2** | | **Regression 3** | | **Regression 4 (expl.)** | | **Regression 5 (sens.)** | |
|  | β ^(adj.)^ | 95%-CI | β ^(adj.)^ | 95%-CI | β ^(adj.)^ | 95%-CI | β ^(adj.)^ | 95%-CI | β ^(adj.)^ | 95%-CI |
| PMB | -0.02 | [-0.11, 0.08] | 0.00 | [-0.09, 0.09] | -0.03 | [-0.13, 0.07] | 0.02 | [-0.07, 0.11] | 0.01 | [-0.08, 0.10] |
| Age |  |  | -0.04 | [-0.13, 0.05] | -0.03 | [-0.12, 0.06] | -0.06 | [-0.15, 0.03] | 0.01 | [-0.08, 0.10] |
| Income |  |  | 0.00 | [-0.08, 0.09] | 0.00 | [-0.09, 0.09] | 0.01 | [-0.07, 0.10] | -0.02 | [-0.10, 0.06] |
| Education |  |  | **0.31^**^** | **[0.12, 0.49]^**^** | **0.29^*^** | **[0.11, 0.48]^**^** | 0.21 | **[0.03, 0.40]^*^** | 0.21 | **[0.04, 0.38]^*^** |
| Non-Heterosexual |  |  | 0.05 | [-0.16, 0.26] | 0.06 | [-0.15, 0.27] | -0.05 | [-0.26, 0.16] | 0.06 | [-0.14, 0.25] |
| MC-SDS |  |  | -0.01 | [-0.10, 0.08] | 0.01 | [-0.08, 0.10] | 0.04 | [-0.05, 0.13] | 0.00 | [-0.09, 0.09] |
| CMNI-30 |  |  |  |  | 0.08 | [-0.02, 0.18] |  |  | 0.08 | [-0.01, 0.17] |
| Emotional Control |  |  |  |  |  |  | -0.09 | [-0.18, 0.00] |  |  |
| Playboy |  |  |  |  |  |  | **0.23^***^** | **[0.14, 0.32]^***^** |  |  |
| Work |  |  |  |  |  |  | -0.02 | [-0.11, 0.06] |  |  |
| Self-Reliance |  |  |  |  |  |  | -0.08 | [-0.17, 0.02] |  |  |
| Relationship |  |  |  |  |  |  |  |  | 0.16 | [-0.03, 0.35] |
| General Health |  |  |  |  |  |  |  |  | 0.09 | **[0.01, 0.18]^*^** |
| Psych. Diagnosis |  |  |  |  |  |  |  |  | 0.00 | [-0.19, 0.18] |
| Freq. Intercourse |  |  |  |  |  |  |  |  | **0.35^***^** | **[0.26, 0.45]^***^** |
| **Model Fit** |  |  |  |  |  |  |  |  |  |  |
| *F* (df_1_, df_2_) | 0.12 (1, 505) | | 1.89 (6, 500) | | 1.97 (7, 499) | | 4.50 (10, 496) | | 11.42 (11, 495) | |
| BIC | 2561.0 | | 2580.9 | | 2584.7 | | 2573.2 | | 2508.8 | |
| Δ*R*^2^ in % | 0.00 | | **1.22^*^** | | 0.28 | | **5.42^***^** | | **17.15^***^** | |
| *Note.* expl. = post-hoc exploratory analyses; sens. = post-hoc sensitivity analyses; β ^(adj.)^ = standardized regression coefficient with significance level adjusted (adj.) for multiple testing using the holm method; 95%-CI = two-sided 95% confidence interval with unadjusted significance level; df = degrees of freedom; Δ*R*^2^ = change in adjusted *R*^2^ for nested consecutive (*R*^2^_1_; *R*^2^_2_ - *R*^2^_1_; *R*^2^_3_ - *R*^2^_2_; *R*^2^_4_ - *R*^2^_2_; *R*^2^_5_ - *R*^2^_3_) models; IIEF = International Index of Erectile Function; PMB = Precarious Manhood Beliefs scale; STIG-9 = Stigma-9 questionnaire; MC-SDS = Marlowe-Crowne Social Desirability Scale; CMNI-30 = Conformity to Masculine Norms Inventory – 30; Psych. = psychiatric; Freq. = frequency. ^*^ *p* < .05; ^**^ *p* < .01; ^***^ *p* < .001 | | | | | | | | | | |

| **Table S5**  *Hierarchical Linear Regression Models 1-5 Predicting Erectile Function (IIEF-EF) with PMB and STIG-9* | | | | | | | | | | |
| --- | --- | --- | --- | --- | --- | --- | --- | --- | --- | --- |
| **Predictor** | **Regression 1** | | **Regression 2** | | **Regression 3** | | **Regression 4 (expl.)** | | **Regression 5 (sens.)** | |
|  | β ^(adj.)^ | 95%-CI | β ^(adj.)^ | 95%-CI | β ^(adj.)^ | 95%-CI | β ^(adj.)^ | 95%-CI | β ^(adj.)^ | 95%-CI |
| PMB | **-0.14^**^** | **[-0.23, -0.04]^**^** | **-0.13^*^** | **[-0.22, -0.04]^**^** | -0.11 | **[-0.21, -0.01]^*^** | -0.09 | [-0.18, 0.01] | -0.07 | **[-0.14, 0.00]^*^** |
| STIG-9 | -0.08 | [-0.17, 0.01] | -0.10 | **[-0.19, -0.01]^*^** | -0.10 | **[-0.19, -0.01]^*^** | -0.10 | **[-0.19, -0.01]^*^** | -0.04 | [-0.10, 0.02] |
| Age |  |  | **-0.21^***^** | **[-0.30, -0.12]^***^** | **-0.22^***^** | **[-0.31, -0.12]^***^** | **-0.22^***^** | **[-0.31, -0.13]^***^** | **-0.16^***^** | **[-0.22, -0.10]^***^** |
| Income |  |  | 0.02 | [-0.07, 0.11] | 0.02 | [-0.06, 0.11] | 0.03 | [-0.06, 0.11] | -0.02 | [-0.07, 0.04] |
| Education |  |  | **0.29^*^** | **[0.11, 0.48]^**^** | **0.30^**^** | **[0.12, 0.49]^**^** | 0.25 | **[0.07, 0.44]^**^** | 0.16 | **[0.04, 0.28]^*^** |
| Non-Heterosexual |  |  | -0.07 | [-0.28, 0.14] | -0.08 | [-0.29, 0.13] | -0.14 | [-0.35, 0.07] | -0.10 | [-0.25, 0.04] |
| MC-SDS |  |  | -0.02 | [-0.11, 0.06] | -0.04 | [-0.13, 0.05] | 0.00 | [-0.09, 0.09] | -0.05 | [-0.12, 0.01] |
| CMNI-30 |  |  |  |  | -0.06 | [-0.16, 0.05] |  |  | -0.06 | [-0.13, 0.01] |
| Emotional Control |  |  |  |  |  |  | **-0.16^**^** | **[-0.25, -0.07]^***^** |  |  |
| Playboy |  |  |  |  |  |  | 0.12 | **[0.03, 0.21]^*^** |  |  |
| Work |  |  |  |  |  |  | -0.08 | [-0.17, 0.01] |  |  |
| Self-Reliance |  |  |  |  |  |  | -0.03 | [-0.12, 0.07] |  |  |
| Relationship |  |  |  |  |  |  |  |  | **0.41^***^** | **[0.27, 0.55]^***^** |
| General Health |  |  |  |  |  |  |  |  | **0.14^***^** | **[0.08, 0.20]^***^** |
| Psych. Diagnosis |  |  |  |  |  |  |  |  | -0.14 | **[-0.27, -0.01]^*^** |
| Freq. Intercourse |  |  |  |  |  |  |  |  | **0.55^***^** | **[0.48, 0.62]^***^** |
| **Model Fit** |  |  |  |  |  |  |  |  |  |  |
| *F* (df_1_, df_2_) | 7.85 (2, 483) | | 6.01 (7, 478) | | 5.40 (8, 477) | | 6.31 (11, 474) | | 61.37 (12, 473) | |
| BIC | 4123.4 | | 4128.9 | | 4133.9 | | 4128.2 | | 3744.6 | |
| Δ*R*^2^ in % | **2.75^***^** | | **3.99^***^** | | 0.03 | | **4.01^***^** | | **53.13^***^** | |
| *Note.* All calculations were performed with the reduced sample (*n* = 486); expl. = post-hoc exploratory analyses; sens. = post-hoc sensitivity analyses; β ^(adj.)^ = standardized regression coefficient with significance level adjusted (adj.) for multiple testing using the holm method; 95%-CI = two-sided 95% confidence interval with unadjusted significance level; df = degrees of freedom; Δ*R*^2^ = change in adjusted *R*^2^ for nested consecutive (*R*^2^_1_; *R*^2^_2_ - *R*^2^_1_; *R*^2^_3_ - *R*^2^_2_; *R*^2^_4_ - *R*^2^_2_; *R*^2^_5_ - *R*^2^_3_) models; IIEF = International Index of Erectile Function; PMB = Precarious Manhood Beliefs scale; STIG-9 = Stigma-9 questionnaire; MC-SDS = Marlowe-Crowne Social Desirability Scale; CMNI-30 = Conformity to Masculine Norms Inventory – 30; Psych. = psychiatric; Freq. = frequency. ^*^ *p* < .05; ^**^ *p* < .01; ^***^ *p* < .001 | | | | | | | | | | |

| **Table S6**  *Hierarchical Linear Regression 1-5 Models Predicting Sexual Desire (IIEF-SD) with PMB and STIG-9* | | | | | | | | | | | |
| --- | --- | --- | --- | --- | --- | --- | --- | --- | --- | --- | --- |
| **Predictor** | **Regression 1** | | **Regression 2** | | **Regression 3** | | **Regression 4 (expl.)** | | **Regression 5 (sens.)** | | |
|  | β ^(adj.)^ | 95%-CI | β ^(adj.)^ | 95%-CI | β ^(adj.)^ | 95%-CI | β ^(adj.)^ | 95%-CI | β ^(adj.)^ | 95%-CI |  |
| PMB | -0.03 | [-0.12, 0.06] | -0.01 | [-0.10, 0.09] | -0.03 | [-0.14, 0.07] | 0.01 | [-0.09, 0.11] | -0.01 | [-0.10, 0.08] |  |
| STIG-9 | 0.02 | [-0.07, 0.12] | 0.00 | [-0.09, 0.10] | 0.00 | [-0.09, 0.09] | 0.01 | [-0.08, 0.11] | 0.03 | [-0.05, 0.12] |  |
| Age |  |  | -0.05 | [-0.15, 0.04] | -0.04 | [-0.14, 0.05] | -0.06 | [-0.15, 0.03] | 0.00 | [-0.09, 0.09] |  |
| Income |  |  | 0.01 | [-0.08, 0.10] | 0.01 | [-0.08, 0.10] | 0.02 | [-0.06, 0.11] | -0.01 | [-0.10, 0.07] |  |
| Education |  |  | **0.32^**^** | **[0.13, 0.51]^**^** | **0.30^*^** | **[0.11, 0.49]^**^** | 0.21 | **[0.02, 0.40]^*^** | 0.22 | **[0.04, 0.39]^*^** |  |
| Non-Heterosexual |  |  | 0.07 | [-0.15, 0.29] | 0.08 | [-0.13, 0.30] | -0.04 | [-0.25, 0.18] | 0.06 | [-0.14, 0.26] |  |
| MC-SDS |  |  | -0.01 | [-0.10, 0.09] | 0.01 | [-0.08, 0.11] | 0.04 | [-0.05, 0.13] | 0.01 | [-0.08, 0.10] |  |
| CMNI-30 |  |  |  |  | 0.08 | [-0.03, 0.18] |  |  | 0.08 | [-0.02, 0.17] |  |
| Emotional Control |  |  |  |  |  |  | -0.10 | **[-0.20, -0.01]^*^** |  |  |  |
| Playboy |  |  |  |  |  |  | **0.24^***^** | **[0.15, 0.33]^***^** |  |  |  |
| Work |  |  |  |  |  |  | -0.03 | [-0.12, 0.06] |  |  |  |
| Self-Reliance |  |  |  |  |  |  | -0.07 | [-0.16, 0.03] |  |  |  |
| Relationship |  |  |  |  |  |  |  |  | 0.18 | [-0.02, 0.38] |  |
| General Health |  |  |  |  |  |  |  |  | 0.10 | **[0.01, 0.19]^*^** |  |
| Psych. Diagnosis |  |  |  |  |  |  |  |  | -0.03 | [-0.22, 0.16] |  |
| Freq. Intercourse |  |  |  |  |  |  |  |  | **0.34^***^** | **[0.24, 0.44]^***^** |  |
| **Model Fit** |  |  |  |  |  |  |  |  |  |  |  |
| *F* (df_1_, df_2_) | 0.23 (2, 483) | | 1.76 (7, 478) | | 1.82 (8, 477) | | 4.37 (11, 474) | | 10.16 (12, 473) | | |
| BIC | 2461.0 | | 2480.1 | | 2484.0 | | 2470.2 | | 2411.9 | | |
| Δ*R*^2^ in % | 0.00 | | **1.40^*^** | | 0.25 | | **6.02^***^** | | **17.14^***^** | | |
| *Note.* All calculations were performed with the reduced sample (*n* = 486); expl. = post-hoc exploratory analyses; sens. = post-hoc sensitivity analyses; β ^(adj.)^ = standardized regression coefficient with significance level adjusted (adj.) for multiple testing using the holm method; 95%-CI = two-sided 95% confidence interval with unadjusted significance level; df = degrees of freedom; Δ*R*^2^ = change in adjusted *R*^2^ for nested consecutive (*R*^2^_1_; *R*^2^_2_ - *R*^2^_1_; *R*^2^_3_ - *R*^2^_2_; *R*^2^_4_ - *R*^2^_2_; *R*^2^_5_ - *R*^2^_3_) models; IIEF = International Index of Erectile Function; PMB = Precarious Manhood Beliefs scale; STIG-9 = Stigma-9 questionnaire; MC-SDS = Marlowe-Crowne Social Desirability Scale; CMNI-30 = Conformity to Masculine Norms Inventory – 30; Psych. = psychiatric; Freq. = frequency. ^*^ *p* < .05; ^**^ *p* < .01; ^***^ *p* < .001 | | | | | | | | | | | |
